# The Association Between Serum MOTS-c Levels and Myocardial Ischemia–Reperfusion Injury in Patients with Acute Myocardial Infarction: A Cross-Sectional Study

**DOI:** 10.3390/biomedicines14040918

**Published:** 2026-04-17

**Authors:** Li Peng, Yanqiu Li, Xinglian Duan, Jun Long, Qin Ran, Xiaojuan Zeng, Bin Liu, Duan Wang, Jian Yang

**Affiliations:** 1 Department of Cardiology, The Third Affiliated Hospital of Chongqing Medical University, Chongqing 401120, China; 651339@hospital.cqmu.edu.cn (L.P.); 651338@hospital.cqmu.edu.cn (Y.L.); 651465@hospital.cqmu.edu.cn (X.D.); 650197@hospital.cqmu.edu.cn (J.L.); 651106@hospital.cqmu.edu.cn (X.Z.); 650648@hospital.cqmu.edu.cn (B.L.); 2 Research Center for Metabolic and Cardiovascular Diseases, The Third Affiliated Hospital of Chongqing Medical University, Chongqing 401120, China; 3 Science and Technology Innovation Center, The Third Affiliated Hospital of Chongqing Medical University, Chongqing 401120, China; ranqin3542@163.com; 4 Department of Clinical Nutrition, The Third Affiliated Hospital of Chongqing Medical University, Chongqing 401120, China

**Keywords:** MOTS-c, acute myocardial infarction, myocardial ischemia-reperfusion injury, percutaneous coronary intervention

## Abstract

**Background/Objectives**: Percutaneous coronary intervention (PCI) effectively restores coronary flow in acute myocardial infarction (AMI), but myocardial ischemia–reperfusion injury (MIRI) remains a major prognostic determinant. Mitochondrial open reading frame of the 12S rRNA-c (MOTS-c) has shown cardiovascular protective effects, yet its association with MIRI is unclear. This study aimed to investigate the relationship between serum MOTS-c levels and MIRI in AMI patients. **Methods**: Seventy-two AMI patients undergoing PCI were enrolled and divided into MIRI (*n* = 34) and non-MIRI (*n* = 38) groups. Clinical data and MOTS-c levels in peripheral serum and intracoronary blood were compared. Multivariate logistic regression and receiver operating characteristic (ROC) analysis were performed to identify MIRI predictors. **Results**: The MIRI group exhibited lower systolic blood pressure, preoperative thrombolysis in myocardial infarction (TIMI) grade, and HDL-C, but higher total ischemic time, door-to-balloon time, culprit vessel stenosis severity, Killip grade and adverse event incidence (all *p* < 0.05). Postoperative peripheral serum MOTS-c levels were significantly lower in the MIRI group than in the non-MIRI group (*p* < 0.05), while preoperative peripheral and intracoronary MOTS-c levels showed no significant differences between groups. Multivariate logistic regression identified postoperative peripheral MOTS-c levels (OR = 0.986, 95%CI: 0.976–0.996) and preoperative TIMI grade ≥ 1 (OR = 0.036, 95%CI: 0.004–0.309) as independent protective factors for MIRI, whereas serum creatinine was identified as an independent risk factor. ROC analysis demonstrated that postoperative peripheral MOTS-c levels predicted MIRI with an area under the curve of 0.648. **Conclusions**: Postoperative peripheral serum MOTS-c levels represent an independent protective factor against MIRI in patients with acute myocardial infarction and suggest a potential predictive value for MIRI, although its clinical utility as a standalone predictor requires further validation through dynamic monitoring and larger-scale studies. This finding may offer a potential novel biomarker and therapeutic direction for MIRI.

## 1. Introduction

Acute myocardial infarction (AMI), a clinical syndrome caused by coronary artery plaque rupture or thrombosis, leads to blood flow obstruction and distal myocardial ischemia [1]. Percutaneous coronary intervention (PCI), one of the main methods for acute myocardial ischemia–reperfusion, promptly opens the infarct-related artery and significantly reduces the mortality rate [2]. However, inevitably, the subsequent myocardial ischemia–reperfusion injury (MIRI) seriously affects the prognosis of patients after PCI [3,4]. Therefore, reduction in the occurrence of MIRI has become an urgent problem to be solved, and searching for prognostic factors related to MIRI has important study value.

Mitochondria are vital functional organelles within cells, serving as the metabolic powerhouses that supply the energy essential for cellular life processes [5]. They also carry genetic materials and possess an independent genetic code—mitochondrial DNA (mtDNA). mtDNA is a double-stranded supercoiled covalently closed circular DNA molecule, which encodes 37 genes, including 22 tRNAs, 2 rRNAs, and 13 mRNAs [6]. Additionally, mtDNA contains small open reading frames (sORFs), which transcribes and translates biologically active short peptides that alter cellular function during stress. These peptides are termed as mitochondrial-derived peptides (MDPs). Currently, identified MDPs include humanin, small humanin-like peptides (SHLPs), and mitochondrial open reading frame of the 12S rRNA-c (MOTS-c) [7]. Research has confirmed that humanin exerts protective effects against diabetes and cardiovascular diseases, primarily through its anti-apoptotic, antioxidant, anti-inflammatory, and mitochondrial function-regulating activities [8,9,10]. Within the SHLPs family, research has predominantly focused on SHLP2 and SHLP3 due to their functional similarities to humanin. Studies confirm that SHLP2 is associated with the progression of age-related diseases, while both SHLP2 and SHLP3 enhance preadipocyte differentiation. They function as insulin sensitizers in peripheral tissues and throughout the body [11].

Mitochondrial open reading frame of the 12S rRNA-c (MOTS-c) is a novel mitochondrial-derived peptide, which exhibits physiological functions similar to but not identical to those of humanin. Studies have shown that MOTS-c exerts many physiological functions, including improving insulin sensitivity, promoting fat and muscle metabolism, promoting the body’s utilization of glucose and fatty acid oxidation, and improving mitochondrial-related functions [12,13]. Serum MOTS-c levels decrease in diabetic and obese patients [10,14]. Additionally, MOTS-c is associated with aging, with its levels declining with increasing age [15]. Given that insulin resistance, diabetes, obesity, dyslipidemia, and aging are all closely linked to coronary heart disease (CHD), the relationship between MOTS-c and CHD has garnered increasing attention. Existing research has demonstrated that MOTS-c possesses cardiovascular protective effects, such as protecting vascular endothelium and inhibiting atherosclerosis [16,17,18]. However, the role of MOTS-c in the regulation of MIRI still remains unknown.

Therefore, this study investigated changes in MOTS-c levels in venous blood and intracoronary blood from patients with AMI undergoing MIRI. We further analyzed the correlation between MOTS-c levels and MIRI to clarify the role of MOTS-c in MIRI. This may provide potential therapeutic targets for treating MIRI.

## 2. Materials and Methods

### 2.1. Study Population

This was a single-center cross-sectional case–control study. A total of 72 patients, who were admitted with AMI in Department of Cardiology, The Third Affiliated Hospital of Chongqing Medical University and underwent PCI from January 2024 to June 2025, were selected. The eligible inclusion criteria included patients who met the diagnostic criteria for AMI and underwent emergency PCI treatment [19]; aged ≥18 years; time from onset to admission < 12 h; and complete clinical documentation. The main exclusion criteria included patients with incomplete clinical data; complicated with severe infection; severe liver and kidney dysfunction; patients with neoplasms; chronic obstructive pulmonary disease; heart valve disorders, congenital heart disease, cardiomyopathy, and endocarditis; and patients with autoimmune diseases or mental disorder. Participants were divided into the MIRI group and the non-MIRI group according to whether MIRI occurred within 48 h after PCI. All subjects signed written informed consent; if the patient was unable to sign, the dependents or guardian signed the written proxy consent. This study was approved by the Ethics Committee of the Third Affiliated Hospital of Chongqing Medical University (Ethics approval number: KELUN 2023 No. 98) and conducted in accordance with the provisions of the Declaration of Helsinki.

MIRI was diagnosed based on established clinical and angiographic criteria [20]. The diagnosis required the presence of at least one of the following after successful coronary recanalization: severe hypotension, bradycardia, or frequent ventricular premature contractions (including accelerated idioventricular rhythm, ventricular tachycardia, ventricular fibrillation, transient sinus bradycardia, sinoatrial block) occur after recanalization of the occluded coronary blood vessels; severe ventricular rhythm disturbance still exists after drug therapy or even electrical cardioversion or defibrillation; coronary angiogram shows that the postoperative coronary antegrade blood flow thrombolysis in myocardial infarction (TIMI) grade is ≤2, without concurrent thrombosis, spasm, or dissection.

### 2.2. Procedures

The clinical data of patients were collected through the electronic medical record system, including gender, age, body mass index (BMI), heart rate, blood pressure, medical history, culprit vessel, Killip grade, TIMI grade, total ischemic time, door-to-balloon time, culprit vessel stenosis severity, medication use, length of hospital stay, and the incidence of adverse cardiovascular events (malignant arrhythmia, cardiogenic shock, sudden death due to left ventricular dysfunction, etc.) during hospitalization was counted. Based on the standard definitions, TIMI flow grade was assessed immediately after final contrast injection following PCI: grade 0, no antegrade flow beyond the occlusion; grade 1, minimal antegrade flow with incomplete distal filling; grade 2, partial antegrade flow with delayed distal filling (typically requiring ≥3 cardiac cycles to achieve distal filling); grade 3, complete antegrade flow with normal distal filling (typically within 3 cardiac cycles). The assessment was performed independently by two interventional cardiologists who were blinded to patient grouping. Any discrepancy was resolved by a third senior cardiologist.

Peripheral venous blood samples were collected from the patients within 6 h after admission. Cardiac troponin I (cTnI) and B-type brain natriuretic peptide (BNP) were detected using a Getein 1600 automatic biochemical analyzer (Getein Biotechnology Co., Ltd., Nanjing, China); left ventricle ejection fraction was measured using a Philips EPIQ5 color ultrasound Doppler diagnostic instrument (Philips (China) Ultrasound Co., Ltd., Shanghai, China). On the next morning after admission, fasting peripheral venous blood was collected from patients, blood lipids [cholesterol total (TC), triglyceride (TG), high density lipoprotein cholesterol (HDL-C), low density lipoprotein cholesterol (LDL-C)], blood urea nitrogen (BUN), serum creatinine (Scr), fasting blood glucose (FBG), hemoglobin glycosylated (HbA1c), and leucocyte count (WBC), etc., were investigated using a Cobas c701 automatic biochemical analyzer (Roche Diagnostics (Shanghai) Co., Ltd., Shanghai, China).

### 2.3. Detection of MOTS-c Levels

Peripheral venous blood samples were collected before PCI and 24 h after the operation. Intracoronary blood samples were obtained from the coronary ostium via the guiding catheter before balloon dilation, as well as immediately after stent deployment and restoration of coronary flow. Coronary sinus sampling, which would reflect outflow from the myocardium, was not performed due to the practical constraints of emergency PCI procedures, where rapid revascularization is the priority. Serum MOTS-c levels were measured using an enzyme-linked immunosorbent assay (ELISA; Chongqing Tairuide Biotechnology Co., Ltd., Chongqing, China). All operations were strictly performed in accordance with the kit instructions.

### 2.4. Statistical Analysis

Count data were expressed as frequencies or percentages (%). Intergroup comparisons were performed using chi-square or Fisher’s exact tests. Ordinal data were analyzed using the rank sum test. Normally distributed continuous data were presented as mean ± standard deviation (SD), and intergroup comparisons were conducted using *t*-tests. Non-normally distributed continuous data were expressed as median (P25, P75), and intergroup comparisons were performed using the Mann–Whitney U test. Multivariate logistic regression analysis was performed to identify factors influencing MIRI after PCI in AMI patients. Receiver operating characteristic (ROC) curves were plotted and area under the curve (AUC) was calculated to assess the predictive value of MOTS-c levels for MIRI occurrence after PCI in AMI patients. All tests were two-tailed, and a *p* < 0.05 was considered statistically significant. Statistical analysis of data was performed using SPSS 26.0 software.

## 3. Results

### 3.1. Comparison of Baseline Data

The baseline data in both MIRI and non-MIRI groups are presented in Table 1. It was found that, compared to subjects in the non-MIRI group, patients in the MIRI group had lower systolic blood pressure and preoperative TIMI grade, but higher total ischemic time, door-to-balloon time, culprit vessel stenosis severity, and Killip grade (*p* < 0.05). Furthermore, the incidence of cardiovascular adverse events during hospitalization was significantly higher in the MIRI group than in the non-MIRI group (52.94% vs. 10.53%, *p* < 0.01). There were 4 patients with congestive heart failure in the non-MIRI group, while 18 patients in the MIRI group had adverse cardiovascular events, including 8 cases of malignant arrhythmia, 11 cases of congestive heart failure, 4 cases of cardiogenic shock and 1 case of death. However, there was no statistically significant difference in other baseline data, including gender composition ratio, age, BMI, history of alcohol consumption, history of smoking, onset time, length of hospital stay, culprit artery, postoperative TIMI classification, use of medication, etc., between the two groups.

### 3.2. Comparison of Inspection Indicators

The inspection indicators in both MIRI and non-MIRI groups were shown in Table 2. Results showed that the HDL-C levels in the MIRI group were significantly lower than those in the non-MIRI group (*p* < 0.05). However, there were no statistically significant differences in other indicators, including creatine kinase–myocardial band (CK-MB), myoglobin, troponin I, B-type natriuretic peptide (BNP), C-reactive protein (CRP), BUN, Scr, TC, TG, LDL-C, HbA1c, fibrinogen (FIB), D-dimer (D-D), left ventricular ejection fractions (LVEF), WBC, uric acid, etc., between the two groups (*p* > 0.05).

### 3.3. Comparison of MOTS-c Levels in Peripheral Venous Blood and Intracoronary Blood Before and After the Operation

**Figure 1:**
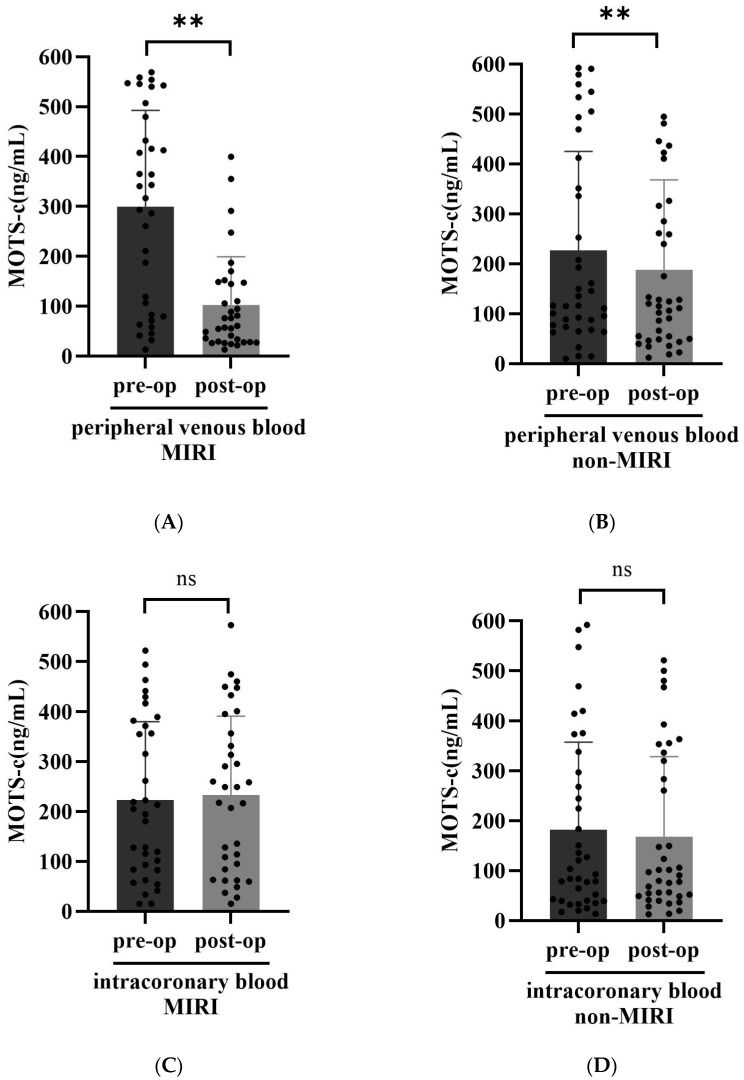
Within-group comparison of MOTS-c levels. Preoperative and postoperative peripheral venous blood MOTS-c levels in the MIRI group (**A**) and non-MIRI group (**B**). ** *p* < 0.01. Preoperative and postoperative intracoronary blood MOTS-c levels in the MIRI group (**C**) and non-MIRI group (**D**). pre-op: preoperative; post-op: postoperative. ns: not significant.

The MOTS-c levels in peripheral venous blood and intracoronary blood before and after the operation were determined. Our results showed that the postoperative peripheral venous blood MOTS-c levels in both MIRI group and non-MIRI group were markedly lower than those of the preoperation (*p* < 0.01) (Figure 1A,B). However, there was no difference in intracoronary blood MOTS-c levels before and after surgery in the two groups (*p* > 0.05) (Figure 1C,D).

**Figure 2:**
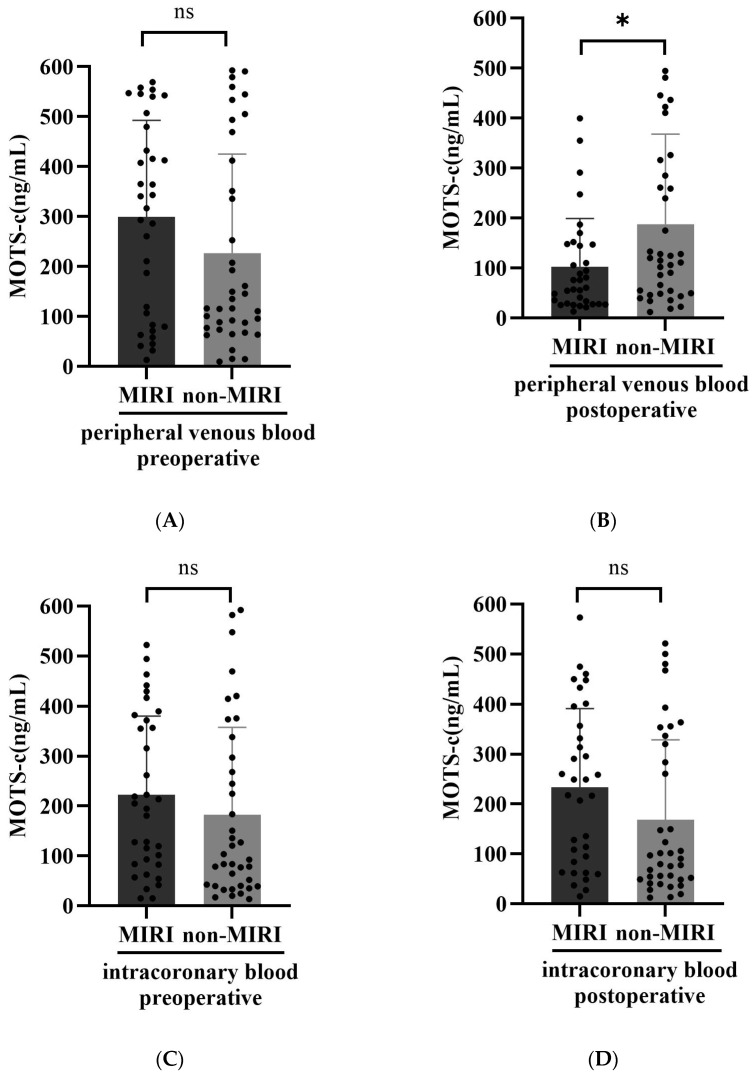
Between-Group Comparison of MOTS-c Levels. Preoperative (**A**) and postoperative (**B**) peripheral venous blood MOTS-c levels between the MIRI group and the non-MIRI group. * *p* < 0.05. Preoperative (**C**) and postoperative (**D**) intracoronary blood MOTS-c levels between the MIRI group and the non-MIRI group. ns: not significant.

Moreover, we also found that the postoperative, not the preoperative, peripheral venous blood MOTS-c levels in MIRI group were significantly lower than those in the non-MIRI group (*p* < 0.05) (Figure 2A,B). Further studies showed that there was no statistically significant difference in intracoronary blood MOTS-c levels before and after surgery between the two groups (*p* > 0.05) (Figure 2C,D).

### 3.4. Independent Factors Associated with MIRI in Patients with AMI

With MIRI as the dependent variable, a multivariate logistic regression analysis was performed using smoking history, history of hypertension, history of diabetes, total ischemic time, door-to-balloon time, culprit vessel stenosis severity, systolic blood pressure, Killip classification (≥grade II = 1, <grade II = 0), preoperative TIMI classification (≥grade 1 = 1, <grade 1 = 0), serum creatinine, HDL-C, random blood glucose, postoperative peripheral serum MOTS-c levels, and intracoronary blood MOTS-c levels after operation. The results indicated that serum creatinine was a risk factor for MIRI, while preoperative TIMI grade ≥ 1 and postoperative peripheral serum MOTS-c levels were protective factors against MIRI (*p* < 0.05, Table 3).

### 3.5. The Predictive Value of MOTS-c for MIRI

**Figure 3:**
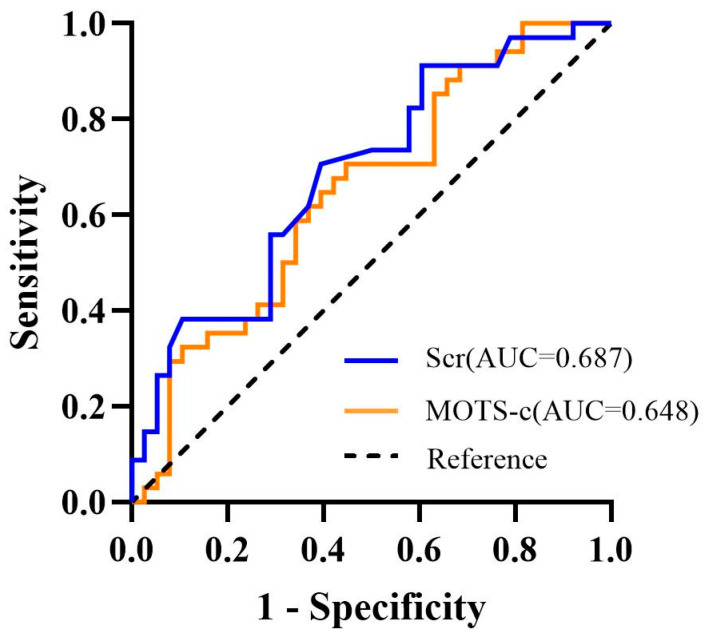
ROC curve of postoperative MOTS-c and serum creatinine for predicting MIRI in AMI patients. ROC, receiver operating characteristic; AUC, area under the curve; Scr, serum creatinine.

ROC curve analysis showed that postoperative peripheral serum MOTS-c and serum creatinine levels have predictive value for the occurrence of MIRI, with areas under the curve (AUC) of 0.648 and 0.687, sensitivities of 0.706 and 0.706, and specificities of 0.553 and 0.605, respectively (Figure 3).

## 4. Discussion

AMI, a critical manifestation of CHD, remains a leading cause of global cardiovascular mortality, accounting for approximately one-third of all deaths worldwide [21]. In China, this burden is particularly substantial because cardiovascular diseases led by CHD are the foremost cause of mortality [22]. Specifically, the incidence of AMI among adults reached 87.6 per 100,000 in 2023 [22], with a mortality rate that continues to rise, especially in rural areas, underscoring its role as a primary lethal outcome of CHD in China [23]. PCI is the primary reperfusion therapy for AMI, aimed at promptly restoring coronary blood flow to reduce mortality. However, the reperfusion process itself can paradoxically induce further myocardial damage, known as MIRI. This injury manifests as reperfusion arrhythmias, myocardial stunning, and cellular necrosis, which collectively diminish the therapeutic benefits of PCI and compromise patient recovery [3,24,25]. Given that MIRI is a key factor limiting the overall efficacy of treatment, mitigating its impact has emerged as an urgent priority in interventional cardiology. Consequently, identifying predictive factors or therapeutic targets associated with MIRI holds significant research value.

MOTS-c, a 16-amino acid polypeptide encoded by the mitochondrial 12S rRNA gene, was discovered in 2015 by Lee et al., using computational methods [26]. This polypeptide is expressed in some organs, including the heart, skeletal muscle, testes, liver, and brain [12]. MOTS-c exerts its effects through autocrine and paracrine mechanisms at both the cellular and systemic levels [27]. In recent years, the potential role of MOTS-c in the cardiovascular system has become increasingly evident. Clinical studies [28] have shown that patients with vascular endothelial dysfunction exhibit lower plasma levels of MOTS-c. Furthermore, MOTS-c levels are positively correlated to endothelial function in both microvessels and epicardial coronary arteries [28]. MOTS-c also improves abnormal blood pressure and echocardiographic parameters, reduces vascular stiffness, maintains cardiac structure, and thereby reverses ventricular remodeling in vitamin D_3_ and nicotine-treated rats [29]. Furthermore, clinical studies revealed that serum MOTS-c levels were significantly lower in acute coronary syndrome patients compared to non-CHD groups, and significantly lower in AMI patients than in unstable angina pectoris patients; moreover, serum MOTS-c levels also decreased significantly with increasing severity of coronary artery lesions, suggesting that low MOTS-c levels may be associated with the onset and progression of CHD [30]. These findings suggest that MOTS-c may play a potential role in the pathophysiology of cardiovascular diseases.

Although the protective role of MOTS-c in cardiovascular diseases has been established, its dynamic changes and prognostic value in the specific pathological process of MIRI remain unknown. Our current study evaluates MOTS-c dynamics in MIRI for the first time. It revealed that peripheral venous blood MOTS-c levels were universally reduced in patients undergoing PCI, with those developing MIRI exhibiting significantly lower postoperative peripheral venous blood MOTS-c levels compared to those without MIRI. However, there were no significant differences in MOTS-c levels in intracoronary blood samples, either within or between groups. This discrepancy between local and systemic levels may, at least in part, be related to sampling time. Intracoronary blood samples were collected immediately after reperfusion, suggesting that dynamic changes in MOTS-c within the coronary circulation might not yet be fully manifested at this early stage. Additionally, our intracoronary sampling was performed at the coronary ostium, which reflects blood entering the coronary circulation rather than draining from the myocardium. Consequently, we were unable to assess the transcardiac gradient of MOTS-c, a more direct measure of myocardial metabolic activity. In the context of emergency PCI for acute myocardial infarction, rapid revascularization is the priority; however, coronary sinus catheterization would prolong procedure time and increase procedural risks, making it challenging to implement routinely in clinical practice. Future studies incorporating sampling from the coronary sinus may help clarify whether local myocardial MOTS-c kinetics differ between groups. However, peripheral venous blood MOTS-c levels may be better to reflect the overall metabolic stress response, as systemic inflammation and metabolic stress are triggered after a period of MIRI. Therefore, the decrease in peripheral MOTS-c levels likely represents pooled changes in its release from multiple organs, including the heart. Multivariate regression analysis further confirmed that postoperative peripheral serum MOTS-c levels and preoperative TIMI grade ≥ 1 constituted independent protective factor against MIRI in AMI patients, while serum creatinine was identified as an independent risk factor.

Our finding extends MOTS-c research field from chronic cardiovascular disease to the realm of acute reperfusion injury in patients. Our results align with a previous basic study concluding that MOTS-c exerts a protective effect against MIRI [31], and are also consistent with reports of Humanin’s cardioprotective role in MIRI [32]. More importantly, patients in the MIRI group exhibited significantly higher rates of major adverse cardiovascular events during hospitalization, including malignant arrhythmia, congestive heart failure, cardiogenic shock, and death. Given that lower postoperative MOTS-c levels were independently associated with increased MIRI risk, and MIRI itself was strongly correlated to adverse clinical outcomes, our findings suggest that MOTS-c may indirectly influence patient prognosis through its association with MIRI. ROC curve analysis showed that postoperative peripheral serum MOTS-c levels demonstrated modest predictive performance for MIRI (AUC = 0.648), indicating limited discriminative ability, and its sensitivity and specificity were suboptimal. This suggests that, as a standalone predictive biomarker, the clinical utility of MOTS-c remains constrained at present. However, as an exploratory study, this current investigation provides the first clinical evidence supporting an association between MOTS-c levels and the risk of MIRI, suggesting its potential as a predictive factor. Nevertheless, the single time-point sampling design limits a comprehensive assessment of its dynamic changes. The modest predictive performance observed in this study may be attributable, at least in part, to the single time-point sampling strategy (24 h post-PCI), which may not have captured the optimal temporal window for MOTS-c dynamics. Future studies incorporating serial time-point measurements (e.g., immediately after PCI, 6 h post-PCI, 24 h post-PCI, and beyond) are warranted to identify the optimal predictive window for MOTS-c. Additionally, combining MOTS-c with other clinical indicators or biomarkers may further enhance its predictive performance.

This study has some limitations. First, as a single-center cross-sectional study, its design can reveal associations but cannot establish causality. Second, the limited sample size may affect statistical power. Third, TIMI myocardial blush grade (TMB), an important indicator of microcirculatory perfusion, was not systematically recorded in this study. This was partly due to the inherent time constraints and procedural prioritization in the emergency PCI setting, as well as the fact that TMB assessment was not incorporated into routine documentation practice at our center during the study period. Fourth, slow-flow phenomenon and thrombus burden grade were not included into the multivariable regression model. According to the MIRI diagnostic criteria, postprocedural TIMI flow grade ≤ 2 is one of the criteria for diagnosing MIRI. Therefore, slow-flow represents an outcome manifestation of MIRI. Including it in the multivariable regression model would create logical overlap with the outcome definition, as it would entail using a component of the diagnostic criteria to predict the outcome itself; therefore, it was excluded. Although thrombus burden grade is associated with postprocedural flow abnormalities; however, according to the diagnostic criteria, patients with postprocedural TIMI ≤ 2 accompanied by significant thrombus could not be diagnosed with MIRI, indicating a distinction between the two types of patients in terms of diagnosis. Given that our current study focused on early periprocedural predictive factors, we prioritized inclusion of variables such as preprocedural TIMI grade and did not include thrombus burden grade in the multivariable model. Fifth, the modest predictive performance of MOTS-c (AUC = 0.648) indicates its discriminative ability is limited, and its sensitivity and specificity were suboptimal; therefore, its clinical utility as a predictive biomarker for MIRI remains limited at present. This may be partly attributable to the single time-point sampling (24 h post-PCI), which precluded dynamic monitoring of MOTS-c changes and may have limited a comprehensive evaluation of its predictive value. Furthermore, the specific molecular mechanisms by which MOTS-c participates in MIRI protection remain unclear. Future studies are needed to validate these findings through larger sample sizes and multi-center prospective cohort studies. Experimental models should be employed to elucidate the mechanism of MOTS-c-mediated effects.

In addition, it should be noted that we opted for conventional logistic regression rather than propensity score matching (PSM) for multivariable analysis, based on the following considerations: (1) In this study, the sample size of the MIRI group (exposed group) was relatively small. Application of PSM would result in further sample size reduction during the matching process. Particularly under caliper matching or 1:1 matching, it would potentially lead to a substantial decrease in sample size after matching, thereby reducing statistical power and possibly failing to detect the true exposure effect. In contrast, conventional logistic regression retains all sample information while controlling for confounders, making it a more appropriate approach in this study. (2) The advantage of PSM lies in balancing measured confounders, but it cannot address unmeasured confounders. In this study, based on univariate analysis, we incorporated clinically recognized potential confounders that differed between groups into the multivariable logistic regression model. This approach effectively controlled for confounding bias while preserving sample size and achieving the goal of adjusting for between-group comparability. (3) The objective of this present study focuses more on estimating “influencing factors” rather than precise causal inference of “treatment effects”. PSM is typically more suitable for estimating treatment effects, whereas the primary objective of this study was to identify independent influencing factors for MIRI. Logistic regression directly provides adjusted odds ratios and confidence intervals for each factor, which aligns better with the objective of this study.

## 5. Conclusions

This study provides the first clinical-level evidence that peripheral venous blood MOTS-c levels following PCI in AMI patients represent an independent protective factor against MIRI and suggest a potential predictive value for MIRI occurrence, although its clinical utility as a standalone predictor requires further validation through dynamic monitoring and larger-scale studies. This finding may offer a potential novel biomarker and potential therapeutic direction for MIRI.

## Figures and Tables

**Table 1:** Comparison of baseline data between MIRI group and non-MIRI group.

Variable	MIRI Group (*n* = 34)	Non-MIRI Group (*n* = 38)	*p* Value
Male sex, *n* (%)	30 (88.2)	29 (76.3)	0.189
Age, years	61.29 ± 12.2	61.92 ± 13.4	0.503
Body mass index, kg/m^2^	24.08 ± 3.02	25.3 ± 3.23	0.640
Heart rate, bpm	80.5 (69.5, 101)	82 (75, 90.25)	0.933
Systolic pressure (mmHg)	126.5 (103, 148)	138 (127, 152.75)	0.029
Diastolic pressure (mmHg)	76.38 ± 16.53	80.66 ± 14.44	0.375
Drink, *n* (%)	11 (32.4)	13 (34.2)	0.867
Smoker, *n* (%)	21 (61.8)	22 (57.9)	0.738
Hypertension, *n* (%)	14 (41.2)	21 (55.3)	0.233
Diabetes mellitus, *n* (%)	7 (20.6)	11 (28.9)	0.413
CHD family history, *n* (%)	1 (2.9)	2 (5.3)	1.000
Angina, *n* (%)	10 (29.4)	18 (47.4)	0.119
Total ischemic time	7.00 ± 4.10	5.01 ± 3.38	0.029
door-to-balloon time	84.68 ± 37.08	65.55 ± 22.62	0.012
culprit vessel stenosis severity	99.47 ± 1.44	98.1 ± 3.1	0.018
LVEF, %	61 (53, 67)	62.5 (57.75, 68)	0.334
Killip grade, *n* (%)			0.020
I	18 (52.9)	32 (84.2)	
II	9 (26.5)	4 (10.5)	
III	3 (8.8)	2 (5.3)	
IV	4 (11.8)	0	
Culprit artery, *n* (%)			0.817
LAD	10 (29.4)	15 (39.5)	
LCX	5 (14.7)	4 (10.5)	
RCA	14 (41.2)	16 (42.1)	
LAD + RCA	1 (2.9)	1 (2.6)	
LCX + RCA	3 (8.8)	2 (5.3)	
LAD + LCX	1 (2.9)	0	
Preoperative TIMI grading, *n* (%)			0.001
0	28 (82.4)	14 (36.8)	
1	2 (5.9)	7 (18.4)	
2	2 (5.9)	13 (34.2)	
3	2 (5.9)	4 (10.5)	
TIMI grading after PCI, *n* (%)			0.174
0	2 (5.9)	0	
1	0	0	
2	1 (2.9)	0	
3	31 (91.2)	38 (100)	
Medication use, *n* (%)			
Aspirin	34 (100)	38 (100)	1.000
Clopidogrel	14 (41.2)	20 (52.6)	0.453
Ticagrelor	20 (58.8)	18 (47.4)	0.331
Statins	34 (100)	38 (100)	1.000
β receptor blocker	23 (67.6)	28 (73.7)	0.574
ACEI/ARB/ARNI	16 (47.1)	25 (65.8)	0.109
Length of hospital stay, days	8 (7, 12.25)	8 (7, 12.25)	0.703
MACE, *n* (%)	18 (52.9)	4 (10.53)	<0.001
Malignant arrhythmia	8 (23.53)	0	
Congestive heart failure	11 (32.35)	4 (10.53)	
Stent thrombosis	0	0	
Cardiogenic shock	4 (11.76)	0	
Death	1 (2.94)	0	

MIRI: myocardial ischemia–reperfusion injury; CHD: coronary heart disease; LVEF: left ventricular ejection fraction; LAD: left anterior descending artery; LCX: left circumflex artery; RCA: right coronary artery; TIMI: thrombolysis in myocardial infarction; PCI: percutaneous coronary intervention; ACEI: angiotensin converting enzyme inhibitor; ARB: angiotensin II receptor blocker; ARNI: angiotensin receptor–neprilysin inhibitor; MACE: major adverse cardiovascular events.

**Table 2:** Comparison of examination indicators between the MIRI group and the non-MIRI group.

Variable	MIRI Group (*n* = 34)	Non-MIRI Group (*n* = 38)	*p* Value
CK-MB (ng/mL)	9.92 (2.5, 30.60)	6.35 (2.66, 25.645)	0.8
MYO (ng/mL)	130.65 (32.02, 288.84)	107.35 (35.27, 155.72)	0.385
cTnI (ng/mL)	0.64 (0.05, 9.63)	0.55 (0.09, 6.55)	0.986
BNP (pg/mL)	72.75 (26.75, 241)	96.1 (29.72, 457.92)	0.531
WBC (109/L)	10.94 ± 3.78	10.18 ± 3.2	0.628
NEUT (109/L)	8.51 ± 3.65	7.8 ± 3.16	0.446
Neu% (%)	75.98 ± 10.23	75.02 ± 11.86	0.500
LYM (109/L)	1.61 (1.135, 2.38)	1.53 (1.07, 2.33)	0.701
HS-CRP (mg/L)	3.20 (1.24, 14.79)	3.23 (1.25, 7.61)	0.839
ALT (U/L)	30 (19.75, 42)	28 (20, 47.75)	0.964
AST (U/L)	52.5 (27.75, 134)	36.5 (22.25, 95.75)	0.219
ALB (g/L)	40.15 (37.85, 43.5)	43.1 (39.52, 44.85)	0.194
Scr (umol/L)	79 (71, 94.5)	76 (59.5, 86.25)	0.086
BUN (mmol/L)	6.20 (5.10, 7.73)	5.52 (4.85, 7.09)	0.388
UA (umol/L)	389.53 ± 96.1	358.39 ± 112.11	0.478
TC (mmol/L)	4.19 (3.54, 5.07)	4.44 (3.85, 5.48)	0.117
TG (mmol/L)	1.68 (1.04, 2.03)	1.27 (1.01, 2.05)	0.580
HDL-C (mmol/L)	0.92 (0.75, 1.15)	1.11 (0.9, 1.33)	0.037
LDL-C (mmol/L)	2.65 (2.11, 3.23)	2.77 (2.27, 3.64)	0.302
FBG (mmol/L)	6.95 (6.27, 8.92)	7.95 (7.02, 11.92)	0.088
HbA1c (%)	6 (5.67, 6.82)	6.2 (5.78, 7.4)	0.387
FIB (g/L)	3.45 (2.84, 4.28)	3.47 (2.74, 3.9)	0.600
D-D (ug/mL)	0.29 (0.14, 0.54)	0.24 (0.12, 0.45)	0.524

CK-MB: creatine kinase–myocardial band; MYO: myoglobin; cTnI: cardiac troponin I; BNP: b-type brain natriuretic peptide; WBC: leucocyte count; NEUT: neutrophil count; Neu%: neutrophil percentage; LYM: lymphocyte count; HS-CRP: high-sensitivity C-reactive protein; ALT: alanine aminotransferase; AST: aspartate aminotransferase; ALB: albumin; Scr: serum creatinine; BUN: blood urea nitrogen; UA: uric acid; TC: cholesterol total; TG: triglyceride; HDL-C: high density lipoprotein cholesterol; LDL-C: low density lipoprotein cholesterol; FBG: fasting blood glucose; HbA1c: glycosylated hemoglobin; FIB: fibrinogen; D-D: d-dimer.

**Table 3:** Multivariate logistic regression analysis of influencing factors for MIRI after PCI in AMI patients.

Variable	B	S.E.	Wald	OR (95%CI)	*p* Value
Smoker	0.511	0.91	0.315	1.667 (0.280–9.925)	0.575
Hypertension	−0.495	0.929	0.284	0.609 (0.099–3.765)	0.594
Diabetes mellitus	1.207	1.773	0.464	3.344 (0.104–107.916)	0.496
Total ischemic time	0.035	0.147	0.056	1.035 (0.776–1.382)	0.813
door-to-balloon time	0.246	0.166	2.186	1.279 (0.923–1.772)	0.139
culprit vessel stenosis severity	0.030	0.018	2.956	1.031 (0.996–1.067)	0.086
Systolic blood pressure	−0.038	0.021	3.073	0.963 (0.923–1.004)	0.080
Scr	0.061	0.028	4.671	1.063 (1.006–1.123)	0.031
HDL-C	0.751	1.667	0.203	2.119 (0.081–55.645)	0.653
FBG	−0.023	0.133	0.030	0.977 (0.752–1.269)	0.862
Killip grade ≥II	1.406	1.190	1.397	4.080 (0.396–42.005)	0.237
Preoperative TIMI grading ≥1	−3.321	1.095	9.204	0.036 (0.004–0.309)	0.002
Postoperative peripheral serum MTOS-c	−0.014	0.005	7.599	0.986 (0.976–0.996)	0.006
Intracoronary blood MTOS-c after operation	0.009	0.005	3.727	1.009 (1.000–1.018)	0.054
Constant	−3.738	4.028	0.861	0.024	0.353

Scr: serum creatinine; HDL-C: high density lipoprotein cholesterol; FBG: fasting blood glucose; TIMI: thrombolysis in myocardial infarction; MTOS-c: mitochondrial open reading frame of the 12S rRNA-c.

## Data Availability

The datasets used and/or analyzed during the current study are available from the corresponding author on reasonable request.

## Abbreviations

The following abbreviations are used in this manuscript:

AMI	Acute myocardial infarction
AUC	Area under the curve
CHD	Coronary heart disease
MDPs	Mitochondrial-derived peptides
MIRI	Myocardial ischemia–reperfusion injury
MOTS-c	Mitochondrial open reading frame of the 12S rRNA-c
mtDNA	Mitochondrial DNA
PCI	Percutaneous coronary intervention
ROC	Receiver operating characteristic
SD	Standard deviation
SHLPs	Small humanin-like peptides
sORFs	Small open reading frames
TIMI	Thrombolysis in myocardial infarction
